# Nitrogenous Fertilizer Coated With Zinc Improves the Productivity and Grain Quality of Rice Grown Under Anaerobic Conditions

**DOI:** 10.3389/fpls.2022.914653

**Published:** 2022-06-28

**Authors:** Muhammad Ashfaq Wahid, Muhammad Irshad, Sohail Irshad, Shahbaz Khan, Zuhair Hasnain, Danish Ibrar, Afroz Rais Khan, Muhammad Farrukh Saleem, Saqib Bashir, Saqer S. Alotaibi, Amar Matloob, Naila Farooq, Muhammad Shoaib Ismail, Mumtaz Akhtar Cheema

**Affiliations:** ^1^Department of Agronomy, University of Agriculture, Faisalabad, Pakistan; ^2^Department of Agronomy, MNS-University of Agriculture, Multan, Pakistan; ^3^National Agricultural Research Centre, Islamabad, Pakistan; ^4^Department of Agronomy, PMAS-Arid Agriculture University, Rawalpindi, Pakistan; ^5^Department of Botany, Sardar Bahadur Khan Women's University, Quetta, Pakistan; ^6^Department of Soil and Environmental Science, Ghazi University, Dera Ghazi Khan, Pakistan; ^7^Department of Biotechnology, College of Science, Taif University, Taif, Saudi Arabia; ^8^University of Sargodha, Sargodha, Pakistan; ^9^School of Science and the Environment, Memorial University of Newfoundland, Corner Brook, NL, Canada

**Keywords:** anaerobic regimes, coated urea, growth, nitrogen, rice, yield

## Abstract

An ample quantity of water and sufficient nutrients are required for economical rice production to meet the challenges of ever-increasing food demand. Currently, slow-release nitrogenous fertilizers for efficient inputs utilization and maximum economic yield of field crops are in the limelight for researchers and farmers. In this study, we evaluated the comparative efficacy of conventional urea and coated urea (zinc and neem) on rice grown under aerobic and anaerobic regimes in greenhouse conditions. For the aerobic regime, field capacity was maintained at 80–100% to keep the soil aerated. On the other hand, for the anaerobic regime, pots were covered with a polythene sheet throughout the experimentation to create flooded conditions. All forms of urea, conventional and coated (zinc and neem), improved plant growth, gas exchange, yield, yield contributing parameters, and quality characteristics of rice crop. However, better performance in all attributes was found in the case of zinc-coated urea. Gas exchange attributes (photosynthetic rate, 30%, and stomatal conductance 24%), yield parameters like plant height (29%), tillers per plant (38%), spikelets per spike (31%), grains per panicle (42%), total biomass (53%), and grain yield (45%) were recorded to be maximum in rice plants treated with zinc-coated urea. The highest grain and straw nitrogen contents, grain protein contents, and grain water absorption ratio were also found in plants with zinc-coated urea applications. In irrigation practices, the anaerobic regime was found to be more responsive compared to the aerobic regime regarding rice growth, productivity, and quality traits. Thus, to enhance the productivity and quality of rice grown in anaerobic conditions, zinc-coated urea is best suited as it is more responsive when compared to other forms of urea.

## Introduction

Among agricultural commodities, rice is a valuable source of food for human beings. It is cultivated in more than 100 countries, with 90% of the total world production from Asia. It is a staple food for more than half of the world's population (Fukagawa and Ziska, 2019). It is listed at the top before all cereals due to its nutrition and dietary value. Ample availability of water and fertilizers are key factors in attaining the optimum yield of rice. Water shortage and rising food demand are significant threats to agriculture. To fulfill the food requirements of the ever-increasing global population, there is a massive increase in demand for rice production (Hillier et al., 2012). However, existing methods of rice production require an abundant supply of water due to the poor water use efficiency. Cultivation of traditional puddled rice is a more common practice in developing countries that can cause a major risk of water scarcity in the long run (Farooq et al., 2007; Punyawattoe et al., 2021). Furthermore, conventional rice production systems (flooded conditions) are time-consuming, water-intensive, tedious, and require enormous amounts of energy to pump out water to maintain the submerged conditions (Mahajan et al., 2012; Rahman et al., 2021). Water-saving technologies for crop production are paramount as the availability of sufficient water plays a significant role in crop productivity (Kazemi et al., 2021) and alters the activities of the rhizosphere in rice (Salsinha et al., 2021). Aerobic rice cultivation, alternate wetting and drying (Beres et al., 2012), persistent soil wetness, and direct (dry) sowing are examples of water-saving technologies in rice production with varying scopes (Cao et al., 2007). Nowadays, researchers are exploring innovative approaches for lowering water use in crop production even while improving the quality of produce. Other benefits of alternate wetting and drying (AWD) include less arsenic absorption by rice grain and reduced water pumping.

Rice is grown in large areas, and it requires a lot of nitrogen (N) each year. A large amount of N fertilizers are lost through volatilization and leaching, thus impacting crop productivity and having a negative effect on the ecosystem. Rice is particularly sensitive to N, and its growth behavior changes significantly in the presence of N fertilizer (Fageria, 2014). Extensive usage of N fertilizers in intensive rice production systems significantly impacts its productivity (Fageria, 2014). Tillage practices, mulching, and integrated nitrogen management are among the approaches developed under the aerobic rice production system to address nitrogen and water losses (Liu et al., 2013). To compensate for N losses and greenhouse gas emissions from paddy fields, slow-release nitrogen fertilizers (SRNF) can be a virtuous choice (Linquist et al., 2012). To mitigate N losses from urea application, manufacturing controlled-release or SNRF is the need of the hour (Zhang et al., 2009). Controlled-release (CR) and/or slow-release (SR) fertilizers are those fertilizers that hold nutrients for a significant interval of time than traditional fertilizers such as urea (Naz and Sulaiman, 2016). Applying mineral elements directly impacts the growth and productivity of rice crops (Herawati et al., 2021).

Farmers are adopting various practices to enhance the productivity of agronomic and horticultural crops (Khasanah and Rachmawati, 2020; Hossain et al., 2021; Rehman et al., 2021), including intercropping, application of mineral elements, synthetic compounds (Afzal et al., 2020; Nurhidayati et al., 2020; Abello et al., 2021), organic amendments (Iqbal et al., 2020; Sarwar et al., 2020; Tabaxi et al., 2021), plants extracts, and biostimulants (Makawita et al., 2021; Khan et al., 2022) *via* soil (Rahim et al., 2020), seed coating (Javed et al., 2021, 2022a), and seed priming agents and foliar spray (Khan et al., 2017; Batool et al., 2019; Farooq et al., 2021). In a study on rice and its response to urea fertilizer amended with bacteria, Fatma et al. (2021) indicated that amended urea was responsible for the variation in the various biochemical activities. Similarly, several strategies are being utilized to improve the efficiency of slow or controlled-release fertilizers. These fertilizers are made by additionally coating functional constituents like paraffin, starch, polythene, and sulpher that hinder the release of nitrogen by creating physical obstruction (Naz and Sulaiman, 2017). Soil enzymes like urea inhibitors are also gaining importance these days to stabilize urea granules (Chagas et al., 2016). Because of reduced soil-related ammonium nitrogen concentrations, CR urea minimizes N leaching and volatilization losses (Li et al., 2017). Different soil parameters like soil moisture, temperature, and environmental variables in the rice-growing areas influence the release pattern of CR urea (Ke et al., 2017). Therefore, it is imperative to assess rice performance in aerobic and anaerobic environments. The high cost of coated urea is the main hurdle preventing its use in agricultural production (Ni et al., 2013). However, coating urea with neem oil or zinc sulfate may have a significant benefit over all other controlled or slow-release urea because of its low input price. Keeping in view the problematic features like intensive water and nitrogen usage for rice production, this study aimed to explore the comparative influence of neem-coated and zinc coated urea with traditional urea on rice growth and productivity under anaerobic rice aerobic regimes.

## Materials and Methods

### Experimental Specifics

The current study planned to explore the influence of urea coated with neem and zinc with traditional urea on the growth, productivity, and quality of rice grown under anaerobic and aerobic regimes. The experiment was conducted in greenhouse conditions at the University of Agriculture, Faisalabad, Pakistan. The germplasm of the newly approved fine rice cultivar of Punjab Basmati was obtained from the Rice Research Institute, Kala Shah Kako, Pakistan. A recommended seed rate of 10 kg per ha was used to raise the nursery on 1 June 2018. For raising the nursery, a wet bed method was used. For this purpose, rice seeds were spread manually on the pulverized soil. For fertilization of the rice nursery, 1.5 kg/6 marla of N was applied. Every morning, the nursery plot was irrigated to a 5 cm depth after seedling establishment. The nursery field was kept free of weeds, insects, and diseases. After 45 days of seedling emergence, the nursery was transplanted to earthen pots filled with soil. The dimensions of the earthen pots were 45 cm in height and 30 cm in diameter. Soil for filling the pots was obtained from the Institute of Soil and Environmental Science, University of Agriculture, Faisalabad, Pakistan. The soil was sandy loam in texture with a pH and EC of 8.21 and 1.3 dS m^−1^, respectively. The organic matter was 0.92% in the soil with nitrogen, phosphorus, and potassium at 580 ppm, 8.9 ppm, and 166 ppm, respectively.


$$\%N=\frac{\;0.0014x\;\left(\;ml\;of\;titration\;for\;sample-ml\;or\;titration\;for\;blank\right)\;x0.1N\;of\;acid\;x250\;\left(d.f\right)}{\;weight\;of\;sample\;\left(g\right)\;x10\;ml\;used\;for\;distillation\;}$$

### Crop Husbandry, Treatment Plan, and Implementation

Irrigation regimes (aerobic and anaerobic) and urea fertilizers (conventional urea, neem-coated, and zinc-coated urea) were the two factors that were focused on in this study. A completely randomized design (CRD) was followed with a factorial arrangement having three repeats. For comparison, we had a control treatment where nitrogen was not applied. Conventional urea, neem-coated urea, and zinc-coated urea were applied at 140 kg per hectare. Conventional urea was applied three times: first, at the time of transplantation (basal dose); second, after 1 month of transplantation; and third, after 2 months of transplantation. Neem-coated and zinc-coated urea were applied as basal doses at the time of transplantation. Potassium and phosphorus fertilizers were also used at 70 and 90 kg per hectare, respectively, as a basal dose. Regarding irrigation regimes, 80–100% field capacity was maintained, and the soil was kept aerated in the aerobic regime. In case of the anaerobic regime, throughout the growing season, a flooded environment was created by covering the pots with polythene sheets. Field capacity was measured according to the gravimetric method described by Nachabe (1998). White, transparent, and 6 mil thickness polythene sheet was used for covering the pots. All other agronomic practices were kept constant for all treatments.

### Estimation of Growth, Yield and Quality Attributes, and Gas Exchange Characteristics

On 15 November 2018, the crop was harvested manually when the grain moisture level was approximately 20–25%. Plants were randomly selected from each experimental unit to record the data on growth, yield, quality attributes, and gas exchange characteristics. During harvesting, plant height was measured from soil base to leaf tip using a measuring rod, and the average was taken. The total number of tillers per plant was counted manually after harvesting, and the mean was calculated thereafter. Grains per spikelet were also counted, and the average was taken. The number of filled and unfilled kernels was counted after harvesting. After measuring the total biomass with the help of an electric weighing balance, samples were threshed to record the weight of the grains. The harvest index was calculated by dividing the grain yield by total biomass and multiplying by 100 for percentage.

Net photosynthesis rate (μmol CO_2_ m^−2^ s^−1^) and stomatal conductance (mol H_2_O m^−2^ s^−1^) were determined using the portable infrared gas analyzer at the reproductive stage. These physiological parameters were recorded of fully expanded youngest leaves from 10:00 to 12:00 h. Kernels were washed with distilled water oven-dried till constant weight was achieved and then, they were ground to a fine powder using a grinder mill (MF 10 IKA, Werkc Germany).

Nitrogen contents were determined by the Gunning–Hibbard method in rice straw and grain.

The Markham Still distillation apparatus was used for distillation, and the nitrogen was determined by the Kjeldahl method. The following formula was used to determine the protein contents:

Grain crude protein contents = N x 6.25,

where 6.25 = constant for wheat according to Bremner and Mulvaney (1982).

Water absorption ratio (WAR) was calculated by dividing the weight of cooked rice by the weight of raw rice, as described by Juliano et al. (1965).

WAR = weight of cooked rice/weight of raw rice.

### Statistical Analysis

Statistical package “Statistix 8.1” was used to analyze the collected data statistically regarding the growth, development, yield, and grain quality. Tukey's HSD test was employed to portray the significant difference in treatments' mean by various alphabets (a, b, c, etc.). For calculation and graphical presentation, Microsoft Excel was used.

## Results

The level of significance of gas exchange attributes, plant growth traits, yield and yield contributing parameters, and grain quality characteristics of rice grown under anaerobic and aerobic regimes in response to coated urea is presented in Table 1. Data regarding plant height indicated a significant effect of fertilizers coated with neem and zinc (Table 2). A significant increase in plant height was observed under varying irrigation regimes along with the application of neem oil and zinc sulfate coated urea, while the interaction of irrigation regimes and fertilizer application was non-significant. Maximum plant height was recorded by applying neem-coated fertilizer, which was statistically at par with zinc-coated fertilizer. Anaerobic irrigation regimes produced longer plants than aerobic irrigation regimes. The highest number of tillers per plant was recorded by application of urea coated with zinc, and the lowest number of tillers were found under control conditions (Table 2). Anaerobic regimes produced more tillers than aerobic irrigation regimes, while their interaction was statistically non-significant. An increase in the number of spikelets per panicle was recorded in crops that received zinc-coated fertilizer, and the lowest number of spikelets was obtained in control. Anaerobic regimes produced the maximum number of spikelets per panicle than aerobic regimes, while the interaction between fertilizer applied and irrigation regimes was non-significant (Table 2). Data regarding the number of filled grains presented in Table 2 show that the application of coated urea fertilizers and different irrigation regimes has a significant effect, while the mutual impact of both factors is not significant. The application of zinc-coated urea was most responsive regarding the filled grains and produced the maximum filled grains. The number of filled grains was reduced in the control, and the anaerobic irrigation regime performed better than the aerobic irrigation regime.

**Table 1:** Mean sum of squares of plant height, tillers, spikelets per panicle, filled grains, unfilled grains, grain yield, total biomass, harvest index, photosynthesis rate, stomatal conductance, nitrogen in straw and grains, protein in grains, and grain water absorption ratio of rice plants grown in aerobic and anaerobic regimes with applications of conventional urea, neem-coated urea, and zinc-coated urea.

**SOV**	**DF**	**Plant height**	**Tillers**	**SPP**	**Filled grains**	**Unfilled grains**	**Grain yield**	**Total biomass**
Irrigation regimes (IR)	1	93.2^*^	6.0**	5.04**	651**	150**	32.6**	92.04**
Urea fertilizer (UF)	3	789**	40**	23.7**	1,726**	243**	106**	1,090**
IR × UF	3	3.28^NS^	1.96^NS^	1.04^NS^	1.04^NS^	12.6^NS^	0.77^NS^	3.71^NS^
**SOV**	**DF**	**HI**	***A***	***gs***	**N in straw**	**N in grains**	**Protein in grain**	**GWAR**
Irrigation regimes (IR)	1	62.3**	9.37**	6402**	0.005^*^	0.041**	0.185^*^	0.326**
Urea fertilizer (UF)	3	598**	46.4**	6,697**	0.225**	0.553**	6.168**	0.370**
IR × UF	3	2.24^NS^	0.37^NS^	222^NS^	0.001^NS^	0.005^*^	0.082^NS^	0.010^NS^

SOV, source of variance; DF, degree of freedom; SPP, spikelets per panicle; HI, harvest index; A, photosynthesis rate; gs, stomatal conductance; N, nitrogen; GWAR, grain water absorption ratio; NS, non-significant, ^*^P ≤ 0.05, P ≤ 0.01.

**Table 2:** Impact of conventional urea, neem-coated and zinc-coated urea on plant height, total number of tillers per plant, number of spikelets per panicle, and number of filled grains per panicle of rice plants grown in aerobic and anaerobic regimes.

**Treatments**	**Plant height (cm)**			**Total tillers per plant**		
	**Aerobic regime**	**Anaerobic regime**	**Mean (UF)**	**Aerobic regimes**	**Anaerobic regime**	**Mean (UF)**
Control	50.10	55.00	52.55 C	9.00	10.00	9.50 D
Urea	63.13	65.10	64.11 B	11.00	12.00	11.50 C
Neem coated	76.33	80.00	78.16 A	13.00	14.00	13.50 B
Zn coated	71.66	76.90	74.28 AB	15.00	16.00	15.50 A
Mean (IR)	65.30 B	69.25 A		12.00 B	13.00 A	
HSD	*IR* = 3.727, *UF* = 10.973, IR × UF = NS			*IR* = 0.672, *UF* = 1.288, IR × UF = NS		
**Treatments**	**Spikelets per panicle**			**Filled-grains per panicle**		
	**Aerobic regime**	**Anaerobic regime**	**Mean (UF)**	**Aerobic regimes**	**Anaerobic regime**	**Mean (UF)**
Control	5.00	6.00	5.50 C	50.00	60.00	55.00 C
Urea	6.00	6.00	6.00 B	70.00	80.00	75.00 B
Neem coated	6.00	6.00	6.00 B	78.33	90.00	84.16 AB
Zn coated	8.00	8.00	8.00 A	90.00	100.00	95.00 A
Mean (IR)	6.25	6.50		72.08 B	82.50 A	
HSD	*IR* = NS, *UF* = 0.392, IR × UF = NS			*IR* = 8.728, *UF* = 16.721, IR × UF = NS		

*IR, irrigation regimes; UF, urea fertilizers; mean values having the same alphabet did not differ significantly at p = 0.05*.

Data regarding unfilled grains per panicle, grain yield per plant, total biomass per plant, and harvest index presented in Table 3 show that the application of coated urea fertilizer and irrigation regimes significantly affected the parameters, though the interaction between them was non-significant. A minimum number of unfilled grains was observed in the case of zinc-coated urea and a maximum number of unfilled grains was observed in the control. Grain filling was highly affected by water stress at the maturity stage. Aerobic regimes increased the number of unfilled grains than anaerobic regimes (Table 3). Maximum grain yield was recorded when fertilizers coated with zinc were used, followed by fertilizers coated with neem. There was a notable reduction in yield in the control, where nitrogen was not used. The anaerobic regime produced the maximum grain yield, followed by the aerobic regime (Table 3). The highest plant biomass was produced when zinc-coated fertilizer was applied, followed by neem-coated fertilizer. Anaerobic conditions produced more biomass than aerobic conditions (Table 3). Data related to harvest index showed a maximum value in the control and a minimum value when fertilizers coated with zinc were used. Anaerobic regimes also had a higher harvest index than aerobic regimes (Table 3).

**Table 3:** Impact of conventional urea, neem-coated urea, and zinc-coated urea on a number of unfilled grains per panicle, grain yield per plant, total biomass per plant, and harvest index of rice plant grown in aerobic and anaerobic regimes.

**Treatments**	**Unfilled grains per panicle**			**Grain yield per plant (g)**		
	**Aerobic regime**	**Anaerobic regime**	**Mean (UF)**	**Aerobic regimes**	**Anaerobic regime**	**Mean (UF)**
Control	28	25	26.5 A	11.00	13.00	12.00 C
Urea	25	16	20.5 B	16.00	18.33	17.16 B
Neem coated	18	14	16.0 C	18.33	20.00	19.16 B
Zn coated	14	10	12.0 D	20.33	23.66	22.00 A
Mean (IR)	21.25 A	16.25 B		16.41 B	18.75 A	
HSD	*IR* = 2.405, *UF* = 2.609, IR × UF = NS			*IR* = 1.468, *UF* = 2.813, IR × UF = NS		
**Treatments**	**Total biomass per plant (g)**			**Harvest index (%)**		
	**Aerobic regime**	**Anaerobic regime**	**Mean (UF)**	**Aerobic regimes**	**Anaerobic regime**	**Mean (UF)**
Control	29.66	31.33	30.50 D	37.08	41.49	39.34 A
Urea	43.00	47.00	45.00 C	37.21	39.0	38.13 B
Neem coated	50.00	55.00	52.50 B	36.66	36.36	36.49 C
Zn coated	60.00	65.00	62.50 A	33.88	36.4	35.2 D
Mean (IR)	45.66 B	49.58 A		36.21 B	37.81 A	
HSD	*IR* = 3.724, *UR* = 7.136, IR × UF = NS			*IR* = 1.084, *UF* = 0.648, IR × UF = NS		

*IR, irrigation regimes; UF, urea fertilizers; mean values having the same alphabet did not differ significantly at p = 0.05*.

Nitrogen (N) concentration in rice straw was significantly affected by fertilizer application and irrigation regimes and their interaction was non-significant (Table 4). The highest concentration of N in rice straw was found by the application of zinc-coated urea, followed by the application of neem-coated fertilizer and a lower concentration of N in straw under control conditions. Anaerobic regimes produced the maximum N concentration in rice straw which was statistically similar to the aerobic irrigation regime (Table 4). Interaction of fertilizers' treatments and irrigation regimes was found statistically significant regarding N concentration in grains (Table 4). The highest N concentration in grains was found where zinc-coated urea was applied under anaerobic conditions. The minimum amount was recorded in control under aerobic conditions, followed by anaerobic regimes (Table 4). Anaerobic irrigation regime had maximum protein contents in grain compared to the aerobic system while the application of coated urea (zinc sulfate and neem oil) also enhanced the protein concentration in grains (Table 4). According to data, different irrigation regimes and N fertilizer had a significant effect on grain water absorption ratio (GWAR), but the interactive effect of both factors was non-significant. Data showed that, among all treatments, the zinc-coated fertilizer was more responsive and produced maximum GWAR. Minimum GWAR was measured in control, while anaerobic conditions also performed comparatively better in the case of GWAR (Table 4).

**Table 4:** Impact of conventional urea, neem-coated urea, and zinc-coated urea on the concentration of nitrogen in straw and grains, protein contents in grain, and grain water absorption ratio of rice plants grown in aerobic and anaerobic regimes.

**Treatments**	**Nitrogen in straw**			**Nitrogen in grain**		
	**Aerobic regime**	**Anaerobic regime**	**Mean (UF)**	**Aerobic regimes**	**Anaerobic regime**	**Mean (UR)**
Control	0.035	0.037	0.036 D	0.89 e	0.95 e	0.92 D
Urea	0.068	0.074	0.071 C	1.00 de	1.16 d	1.08 C
Neem coated	0.123	0.128	0.126 B	1.36 c	1.40 bc	1.38 B
Zn coated	0.445	0.456	0.450 A	1.57 ab	1.63 a	1.60 A
Mean (IR)	0.168 A	0.174 A		1.20 B	1.28 A	
HSD	*I* = 0.007, *F* = 0.674, *IF* = NS			*I* = 0.053, *F* = 0.01, *IF* = 0.017		
**Treatments**	**Protein in grain**			**Grain water absorption ratio**		
	**Aerobic regime**	**Anaerobic regime**	**Mean (UF)**	**Aerobic regimes**	**Anaerobic regime**	**Mean (UR)**
Control	5.80	6.00	5.90 C	2.03	2.33	2.183 B
Urea	6.30	6.16	6.23 C	2.20	2.40	2.30 B
Neem coated	7.20	7.40	7.30 B	2.43	2.56	2.50 AB
Zn coated	7.90	8.33	8.11 A	2.60	2.90	2.75 A
Mean (IR)	6.80 B	6.97 A		2.31 B	2.55 A	
HSD	*I* = 0.418, *F* = 0.674, *IF* = NS			*I* = 0.173, *F* = 0.331, *IF* = NS		

*IR, irrigation regimes; UF, urea fertilizers; mean values having the same alphabet did not differ significantly at p = 0.05*.

**Figure 1:**
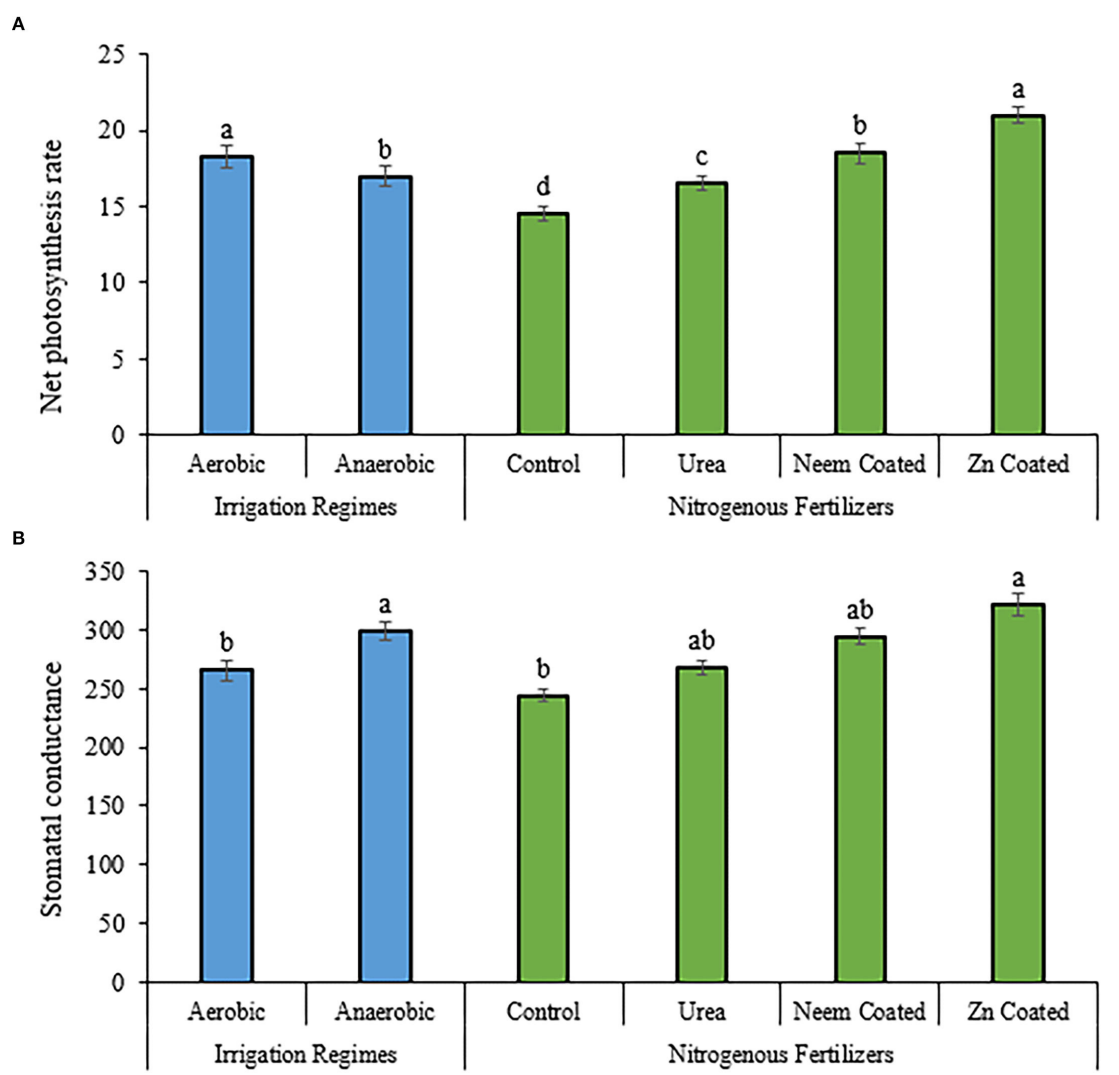
Influence of slow release nitrogenous fertilizers on mean values of net photosynthesis rate **(A)** and stomatal conductance **(B)** of rice cultivated under aerobic and anaerobic environment.

The photosynthetic rate was significantly affected by fertilizer application and irrigation regimes though the interaction was non-significant (Figure 1). The highest photosynthetic rate was observed in the case of application of urea coated with zinc, which was followed by the application of neem-coated fertilizer, while aerobic condition performed better than anaerobic condition. Data related to stomatal conductance, presented in Figure 1, shows that the application of zinc-coated urea produced more value. Stomatal conductance was also higher in anaerobic regimes compared to aerobic regimes.

## Discussion

Nitrogen (N) is an essential macronutrient that enhances vegetative growth in plants. Nitrogen regulates the physiological and morphological processes in crops, so a proper dose of N is considered beneficial for the optimum growth of plants. According to our findings, accessibility of N was more in coated urea (CU) treatments than in any other treatment. Increased plant height was particularly due to a better stand establishment, cell division, and proper absorption of nutrients from the soil. Cassman et al. (2003) reported that nitrogen availability throughout the growing season was more in coated urea than in other urea sources. As rice is a hydrophilic crop, plenty of water was made available throughout the growing period under anaerobic conditions that may have resulted in increased plant height. Our results are similar to those reported by Belder et al. (2007). Wu et al. (2017) observed taller maize plants under controlled-release urea (CRU) than chemical fertilizer (CF) treatment. The application of nitrogenous fertilizers improved the growth of maize plants cultivated even under unfavorable conditions (Imran et al., 2021). Javed et al. (2022b) also reported that the application of various organic and inorganic compounds was responsible for improving nitrogen use efficiency. Khan et al. (2021) stated that nitrogen application enhanced the growth and productivity of field crops and maintained the soil fertility status (Shah et al., 2021a).

The number of tillers per plant is the main feature to produce more grains and thereby a vital aspect of crop productivity. Soil water and nutrient availability may become limiting factors when rice is cultivated aerobically. Nutrient movement through mass flow and diffusion slows down as soil moisture reduces (Marschner, 1995). Therefore, continuous N supply can enhance tiller development. Nitrogen uptake in flooded conditions increased four-fold compared to uptake in aerobic conditions (Bouman and Tuong, 2001). The findings of the current study are also in line with the research of Qamar et al. (2020), who concluded that the application of mineral elements improved the growth attributes of wheat crops. Similar effects were also observed by Nie et al. (2012). Golden et al. (2009) reported the same results for coated urea, where the nitrogen use efficiency (NUE) from conventional urea was 12-15% lower than CRU as a single basal dose. Yang et al. (2012) stated that the NUE of CRU was 27.6 and 22.9% higher than those of conventional urea applied at a rate of 300 kg N ha^−1^ in 2007 and 2008, respectively. An approximate increase of 25% NUE was reported with polymer-coated urea (PCU) applications compared to straight urea by Fageria and Carvalho (2014). Tanga et al. (2020) and Kumar et al. (2022) stated that the application of mineral elements significantly enhanced the nutrient use efficiency, growth, and biochemical attributes of field crops with a maximum net return (Yousaf et al., 2016; Shah et al., 2021b).

Findings of the current study show that kernel yield under CRU was higher than conventional urea. A sufficient supply of water during AWD could expand root growth, assist the translocation of C reserves to the grains, enhance grain filling, and improve grain yield. Continuous supply of N produced more number of productive tillers, which increased the grain-filled process of the rice crop. Nitrogen application encouraged a sink capacity (the number of spikelets per panicle), whereas it squeezed the dry matter translocation rate (from source to sink), which resulted cumulatively in reducing grain filling for the secondary and tertiary tillers (Puteh et al., 2014). Growth period and culm length had reduced in inferior tillers in the study by Wang et al. (2016). Shading and premature senescence might be critical factors, apart from source limitations, to further reduce the grain filling percentage of mediocre tillers (Mo et al., 2015). Past studies reveal that grain weight was the most vital yield component, less affected by environmental factors, and was a highly genetic character of the crop (Guo et al., 2009). Low N concentration in conventional urea reduced the grain weight. Application of coated urea fertilizers and adequate moisture enhanced the number of superior tillers that increased the grain weight and enhanced the number of filled grains. Moreover, the lesser grain weight of rice could be the consequence of metabolic disorders relating to carbon and N under stress conditions, especially water stress (Jiang et al., 2016).

Nitrogen fertilizers reduce the number of unfilled grains under anaerobic conditions at outstanding levels because panicle sterility is among the major constraints of the aerobic rice system (Farooq et al., 2011). Nitrogen and potassium application reduces panicle sterility in rice (Awan et al., 2007). Previous studies confirmed that foliar application of KNO_3_ improved the paddy yield by diminishing panicle sterility (Mahajan and Khurana, 2014). Because of the reduced total number of unfilled grains, the total number of filled grains enlarged when N fertilizer was applied. Nitrogen shortage produced more sterile pollen in rice, which in turn reduced the number of normal kernels (Esfehani et al., 2005). Hayashi et al. (2008) evaluated that the application of methylene urea (MU) enhanced root surface area in rice, which in turn provided a favorable environment for the uptake of nutrients like N. The activity of glutamine synthetase (GS) and nitrate reductase (NR) in rice leaves was higher in polymer-coated urea than in CU. The activity of GS and NR in maize was higher in MU than in CU. A higher NR and GS activity indicates greater synthesis of protein, greater N assimilation, and better plant growth, which in turn will enhance grain filling and reduce the percentage of unfilled grains (Geng et al., 2016). The findings of our study are also supported by Zahid et al. (2021), who reported that the application of urea coupled with poultry manure was responsible for the improved growth, yield, and quality of cucumber.

Dong et al. (2012) reported that straw biomass was affected by two different irrigation regimes. A highly positive relationship was shown between panicle biomass and grain yield. Leaf and stem biomass did not differ between AWD and continuous flooding, but it was notably enlarged with an application of diverse N fertilizers. At maturity, AWD produced more yield than CF when the same amount of N fertilizer was applied. Gong et al. (2005) proposed that the ample amount of soil water before maturity in CF was a major factor in enhancing shoot and total plant biomass during the anthesis and booting stages. During grain filling and maturity stages, PCU was favorable for rice growth, as reported by Zhang et al. (2011). Their study explained that plants under polymer-coated urea treatment had expressively higher plant biomass than conventional urea applied after the heading stage. Both coated urea and AWD management significantly enhanced total biomass production as a result of better root growth (Mahajan et al., 2012), which also led to the development of a better canopy as interception of light was more (Yao et al., 2012). Huang et al. (2022) stated that the application of nitrogen improved the antioxidant activities linked with better growth of *Wedelia trilobata*.

Among other physiological processes, photosynthesis is inhibited by water stress (Wingler et al., 1999). During early growth stages, mild water stress can cause stomatal closure (Chaitanya et al., 2003), which declines the photosynthetic rate (Flexas et al., 2006). However, our findings proved that the rate of photosynthesis in rice was not affected significantly by the interaction of both factors (CU × I). Stomatal closure induced by lower water availability may be a dynamic influence on plant photosynthesis. Application of N normally resulted in an increased amount of Rubisco and leaf N concentration, as reported by Ookawa et al. (2004). The findings of our experiment is in line with the outcomes of Wang et al. (2022), where the application of nitrogenous fertilizers in integration with straw improved the photosynthetic activities and growth parameters in winter wheat. Larger concentrations of Rubisco in photosynthesis were moderately compensated by low Rubisco activation, which could explain why the N application rate may not increase photosynthesis at a significant level (Ray et al., 2003). Limited water supply to plant reduced total N content and transpiration rate in the shoots of rice plant. The amount of transpired water was reduced due to impeded shoot growth as a result of water stress. Several other reports described that reduction in LNC induced stomatal closure and gs depression, through which the net photosynthetic rate (PN) of rice leaf was reduced. A shortage of water could reduce enzyme activity, photosynthetic rate, uptake of nutrients, and membrane fluidity (Xu et al., 2015). Nitrogen application levels also affected plant growth in our experiment. During the heading and grain filling stage, water-saving unfavorably reduced rice leaf area, plant height, and biomass at (N_0_) and (N_1_) treatments. We proposed that reduced rice biomass may be a consequence of reduced leaf area and plant height, which pointedly decreased the UE of light energy under water-scarce conditions (Yang and Zhang, 2010).

Many past studies demonstrated that, at maturity, most of the N translocated to panicle previously found in leaves and stem as shown by the dying and yellowing of the older leaves during harvest. Similar results were determined by Sariam (2004), who reported that more N accumulation from zinc-coated urea may also be owed to the deliberate release of N throughout the growing seasons. Lowest N loss through ammonia volatilization and better N availability through the controlled release of urea fertilizers throughout the growing season with a steady discharge of N to the soil have the capacity to improve N uptake. Similar results were found by Vimala and Subramaniam (1994). Total N concentration in grains has enlarged with the application of CRU. According to these effects, controlled-release urea fertilizer (CRUF) had a more encouraging effect than conventional urea. Plant population had a minimum effect on kernel quality. Whereas, N application had an imperative effect on pollination and fertilization. Improved translocation of photo-assimilates and N toward the panicle with a slow-release supply of N at anthesis enhanced the healthy kernels and reduced chalky, unfilled, and opaque kernels in the flooded system than in the aerobic system. Ahmad et al. (2009) provided corresponding findings to our study and reported that kernel quality improved when the nitrogen supply was optimum irrespective of plant population and density. The outcomes of the present experiment are also supported by Huang et al. (2022), who concluded that the application of nitrogenous fertilizer enhanced the antioxidant activities and growth attributes in *Wedelia trilobata*.

## Conclusion

The current experiment was designed to study the comparative impact of traditional urea with zinc-coated and neem-coated urea on the growth and productivity of rice cultivated under aerobic and anaerobic regimes. All forms of urea significantly improved the gas exchange attributes, productivity, and quality parameters. However, the application of zinc-coated urea was found most responsive to improving the quality and productivity of rice plants. Grain yield and total biomass were also recorded as maximum in response to the application of zinc-coated urea. Nitrogen concentration in rice straw and grains was also increased by applying zinc-coated urea. The anaerobic regime was found more responsive compared to the aerobic regime regarding rice growth, productivity, and quality of produce. Further research is required to explore the physiological and biochemical mechanisms of zinc-coated urea regarding the enhancement in agronomic and quality attributes of rice.

## Data Availability Statement

The original contributions presented in the study are included in the article/supplementary material, further inquiries can be directed to the corresponding authors.

## Author Contributions

All authors listed have made a substantial, direct, and intellectual contribution to the work and approved it for publication.

## Conflict of Interest

The authors declare that the research was conducted in the absence of any commercial or financial relationships that could be construed as a potential conflict of interest.

## Publisher's Note

All claims expressed in this article are solely those of the authors and do not necessarily represent those of their affiliated organizations, or those of the publisher, the editors and the reviewers. Any product that may be evaluated in this article, or claim that may be made by its manufacturer, is not guaranteed or endorsed by the publisher.
